# Recruitment of veterans from primary care into a physical activity randomized controlled trial: the experience of the VA-STRIDE study

**DOI:** 10.1186/1745-6215-15-11

**Published:** 2014-01-07

**Authors:** Marquis S Hawkins, Linda J Hough, Marie A Berger, Maria K Mor, Ann R Steenkiste, Shasha Gao, Roslyn A Stone, Kelly H Burkitt, Bess H Marcus, Joseph T Ciccolo, Andrea M Kriska, Deborah T Klinvex, Mary A Sevick

**Affiliations:** 1 Division of Biostatistics & Epidemiology, University of Massachusetts, Amherst School of Public Health and Health Sciences, Amherst, MA 01003, USA; 2 VA Pittsburgh Healthcare System, Center for Health Equity Research and Promotion, Pittsburgh, PA 15206, USA; 3 Department of Biostatistics, University of Pittsburgh Graduate School of Public Health, Pittsburgh, PA 15260, USA; 4 Department of Family and Preventive Medicine, University of California San Diego, La Jolla, CA 92093, USA; 5 Department of Biobehavioral Sciences, Columbia University Teachers College, New York, NY 10027, USA; 6 Department of Epidemiology, University of Pittsburgh Graduate School of Public Health, Pittsburgh, PA 15260, USA; 7 University of Pittsburgh Clinical and Translational Science Institute, Pittsburgh, PA 15260, USA; 8 Department of Population Health, New York University School of Medicine, New York, NY 10016, USA

**Keywords:** Veterans, Primary care, Physical activity, Overweight, Randomized controlled trial

## Abstract

**Background:**

Much of the existing literature on physical activity (PA) interventions involves physically inactive individuals recruited from community settings rather than clinical practice settings. Recruitment of patients into interventions in clinical practice settings is difficult due to limited time available in the clinic, identification of appropriate personnel to efficiently conduct the process, and time-consuming methods of recruitment. The purpose of this report is to describe the approach used to identify and recruit veterans from the Veterans Affairs (VA) Pittsburgh Healthcare System Primary Care Clinic into a randomized controlled PA study.

**Methods:**

A sampling frame of veterans was developed using the VA electronic medical record. During regularly scheduled clinic appointments, primary care providers (PCPs) screened identified patients for safety to engage in moderate-intensity PA and willingness to discuss the study with research staff members. Research staff determined eligibility with a subsequent telephone screening call and scheduled a research study appointment, at which time signed informed consent and baseline measurements were obtained.

**Results:**

Of the 3,482 veterans in the sampling frame who were scheduled for a primary care appointment during the study period, 1,990 (57.2%) were seen in the clinic and screened by the PCP; moderate-intensity PA was deemed safe for 1,293 (37.1%), 871 (25.0%) agreed to be contacted for further screening, 334 (9.6%) were eligible for the study, and 232 (6.7%) enrolled.

**Conclusions:**

Using a semiautomated screening approach that combined an electronically-derived sampling frame with paper and pencil prescreening by PCPs and research staff, VA-STRIDE was able to recruit 1 in 15 veterans in the sampling frame. Using this approach, a high proportion of potentially eligible veterans were screened by their PCPs.

**Trial registration:**

Clinical trials.gov identifier: NCT00731094.

## Background

Physical inactivity is widely known to be associated with many diseases, including diabetes and cardiovascular disease, and yet less than 46% of US adults met recommendations for aerobic physical activity (PA) in 2010 [1]. To increase adherence to these guidelines, evidence-based intervention approaches are needed. Much of the existing literature on PA interventions involves physically inactive individuals recruited from community settings rather than clinical practice settings. However, 83% of adults see a health care provider annually [2], and are receptive to advice from their clinicians on making lifestyle change [3]. Consequently, primary care may be an ideal location to implement PA intervention programs.

Recruitment of patients into interventions from clinical practice settings is difficult due to limited time available in the clinic and identification of appropriate personnel to efficiently conduct the recruitment process. While involving clinicians in recruiting patients into randomized controlled trials can substantially increase referral and enrollment rates [4,5], this method can be time consuming, costly, and burdensome to clinic staff and providers [6]. Failure to address these barriers can hamper study recruitment, and threaten external validity [7].

To address barriers of clinician recruitment, numerous automated methods have been used to identify potentially eligible participants [4,8-12]. While these methods have enhanced recruitment, alert fatigue and dismissal of auto alerts may reduce screening of potentially eligible participants. Other strategies are needed to improve recruitment efforts.

The purpose of this report is to describe: (1) the approach used to recruit potentially eligible veterans from the Veterans Affairs (VA) Pittsburgh Healthcare System University Drive Division Primary Care Clinic (VAPHS UD) into a PA intervention trial, (2) adherence by primary care providers (PCPs) to the recruitment protocol, (3) reasons for non-enrollment of veterans, and (4) recruitment yields.

## Methods

### Design

VA-STRIDE was a randomized controlled clinical trial designed to evaluate the effectiveness and cost utility of a theory and expert system-based PA intervention to deliver individually tailored motivational messages to veterans who were physically inactive (that is, those who reported less than 60 minutes of moderate PA, such as brisk walking, per week) and overweight or obese [3,10,13]. Veterans who received routine care through VAPHS UD were screened and recruited using a four-part approach (described below). Those randomized to the intervention group received 12 months of the standardized intervention. Those randomized to the attention control group received one face-to-face counseling session about healthy lifestyle behavior from a health educator and monthly wellness mailings focusing on health but not PA. Both groups received 12 months of routine primary care through VAPHS UD. Recruitment for the study began in June 2010 and ended in April 2012, with the final measurement visits completed in March 2013. Recruitment eligibility criteria, methods, and forms remained the same throughout the study following minor edits of a screening form in the first month after consulting with PCPs about ease of use.

This study was approved by the VAPHS Institutional Review Board (IRB), protocol #02702. Signed informed consent was obtained from all participants.

### Sample

The recruitment strategy was designed to enroll 300 physically inactive and overweight or obese veterans who could safely participate in self-directed PA of at least moderate intensity on most days of the week. We sought to enroll veterans for whom PCPs could recommend unsupervised moderate-intensity PA as part of routine care without requiring additional safety testing.

To be included in the sampling frame for the study, the veteran: (1) had one or more primary care clinic visits at the VAPHS UD in the 12 months before enrollment, (2) was at least 18 years of age at the time of enrollment, and (3) was overweight or obese with a body mass index (BMI) ≥25 kg/m^2^ calculated based on the most recent height and weight recorded in the medical record. Because we sought a representative sample of the VAPHS veteran population, exclusion criteria were limited to factors that would limit safety, feasibility, or potential to benefit from the intervention.

Excluded from the sampling frame were veterans with existing *International Classification of Diseases*, ninth edition (ICD-9) codes for psychoses (codes 290 to 299), alcohol or drug dependence (codes 303 and 304), mental retardation (codes 317 to 319), unstable angina (code 411.1), pulmonary hypertension (codes 416.0, 416.8), spinal cord injuries (codes 806 and 952), and long-term oxygen therapy (code V46.2). Because the intervention advised brisk walking, veterans in wheelchairs (code V53.8) or who required assistive walking devices (for example, canes and walkers), and those who could not walk at least 120 yards unassisted were excluded from the study. Because this was a print-based intervention and available only in English, with materials distributed to veterans via US postal mail, participants were required to read English at a 7th grade level or above. We also excluded those who reported they already participated in moderate intensity activities for at least 60 minutes per week, lived in an institutional setting, planned to move out of the VAPHS service area in the next 12 months, were employees of VAPHS, expressed unwillingness to adhere to the study protocol, or were participating in other clinical studies that would be expected to have an impact on PA.

### Recruitment

We established an electronic sampling frame using clinical data recorded in the Veterans Health Information Systems and Technology Architecture (VistA), the information system built around an electronic health record that is used throughout the Veterans Health Administration. VistA provides an integrated inpatient and outpatient electronic record that supports VA clinical and administrative functions. We abstracted from VistA the records of Veterans receiving primary care at VAPHS UD after 1 June 2009. Subject-specific data were used to identify potentially eligible veterans according to the criteria described above.

To identify PCPs to participate in the screening process, we engaged the Medical Directors of the Primary Care Clinics to introduce the principal investigator (PI) and research staff to PCPs during weekly meetings. At that time, a very brief overview of the study was provided and PCPs were told that our goal was to recruit veterans for whom they would be comfortable recommending unsupervised moderate-intensity PA, comparable to brisk walking, as part of routine care and without the need for further safety testing. The PCPs also were shown screening form I (SF-I; see Additional file 1), which they were asked to complete during the veteran’s clinical visit as described below. PCPs participating in the screening process were those who were informed about the research study, were instructed in completing the screening form, and agreed to screen their patients.

Research staff identified the sampling frame electronically from the VistA records with a database query for age, calculated BMI, primary care history, presence of exclusionary ICD-9 codes, and PCP willingness to participate. This was performed at the beginning of each week to identify specific appointments for the following 5 days. Those patients who visited VAPHS for the first time during the recruitment period or had changes to their BMI or ICD-9 codes were included in subsequent database queries.

Research staff generated the patient-specific SF-I the day before each potentially eligible veteran’s scheduled appointment. The SF-I was prepopulated automatically with the veteran’s preassigned study ID number and the name of the PCP. Research staff attached the SF-I and a one-page flier describing the study to a routinely provided list of the veteran’s medications that PCPs were to review during the appointment. Clinic staff put the forms in a receptacle next to the exam room to be retrieved by the PCP when entering the room. The SF-I was printed on neon-green paper to make it noticeable to clinic staff and PCPs.

The second part of the recruitment process involved PCP screening using the SF-I. The PCP was asked to respond to the question: 'Given your knowledge of this patient, can this Veteran SAFELY do progressive, unsupervised physical activity at home (regardless of his/her interest in doing so)? Participants may be asked to work up gradually to 30 minutes of moderate physical activity (e.g., brisk walking, swimming) on most days of the week’. Response categories were 'no/not sure’ or 'yes’, further specifying with or without restrictions (Additional file 1). Defining the restrictions was left to the discretion of the PCP, to streamline the form and encourage PCPs to complete it.

Next, the PCP gave the one-page flier to the veteran and asked permission for research study staff to contact him/her directly to discuss possible enrollment in VA-STRIDE ('yes/no’). Lastly, the PCP signed and returned the form to the patient to give it to the clinic clerical staff at checkout, regardless of the veteran’s eligibility or willingness to be contacted. A unit clerk then detached the SF-I form from the clinical paperwork and filed the form in a secure location for later retrieval by research staff. In the event they were too busy to screen a veteran, PCPs were given the option to return a blank SF-I form to the research staff. When this occurred, the unscreened veteran was returned to the sampling frame for consideration at a subsequent primary care appointment. If a participating PCP demonstrated low rates of recruitment or incorrect completion of SF-I, details of the screening approach were reinforced by the PI or research staff during a subsequent face-to-face discussion. To acclimate PCPs to this process, we capped the number of SF-Is generated to five patients per PCP per day in the initial recruitment period. As PCPs became familiar with the study screening process, the screening cap was lifted. Screening was halted occasionally to accommodate staff availability. When screenings were limited or halted, veterans not approached were retained in the sampling frame for consideration at a subsequent primary care visit. The veteran was also returned to the sampling frame if he or she was not seen in clinic (that is, cancellation or 'no show’), the SF-I was not returned (that is, the patient left the clinic without turning in the form), or the SF-I was returned blank (that is, the PCP did not complete the screening).

During the third part of the recruitment process, veterans who gave their permission were then contacted via telephone by research staff to administer screening form II (SF-II; see Additional file 2) to confirm information pertaining to study exclusion criteria and the veteran’s ability to safely participate in the intervention. Waivers of Health Insurance Portability and Accountability Act (HIPAA) authorization and documented informed consent for screening and completion of SF-II were granted by the VAPHS IRB to minimize disruptions to the general functioning of the primary care clinic and eliminate response burden for those patients who would not be eligible for the study.

A brief overview of the study was provided to patients prior to administration of the SF-II to inform them that participants in both study groups would have a series of 14 mailings to their homes, including 9 PA questionnaires mailed to the intervention group. Participants in the intervention group would receive follow-up feedback reports based on responses to the questionnaires and generated by the expert system. Length of participation would be 48 weeks (defined as 12 months), and monetary compensation for time and travel would be $25 for each of the three measurements visits (baseline, 6 months, and 12 months), and $15 for the one orientation counseling visit following the baseline visit.

After the SF-II was completed, veterans who remained eligible and had continued interest in the study were scheduled for a baseline appointment. Research staff obtained signed informed consent, followed by baseline measurements at the first study visit. Veterans were randomized to either the intervention or attention-control group and scheduled for an orientation counseling session pertinent to their assignment. The veterans were informed of their study group assignment only at the counseling session.

Measurements were completed at baseline, 6, and 12 months for the primary outcome (that is, amount of PA per week) and secondary outcomes (that is, fitness for PA; health-related quality of life; weight, body composition, blood pressure, inflammation, glucose metabolism, and serum lipids; and costs).

## Results

**Figure 1:**
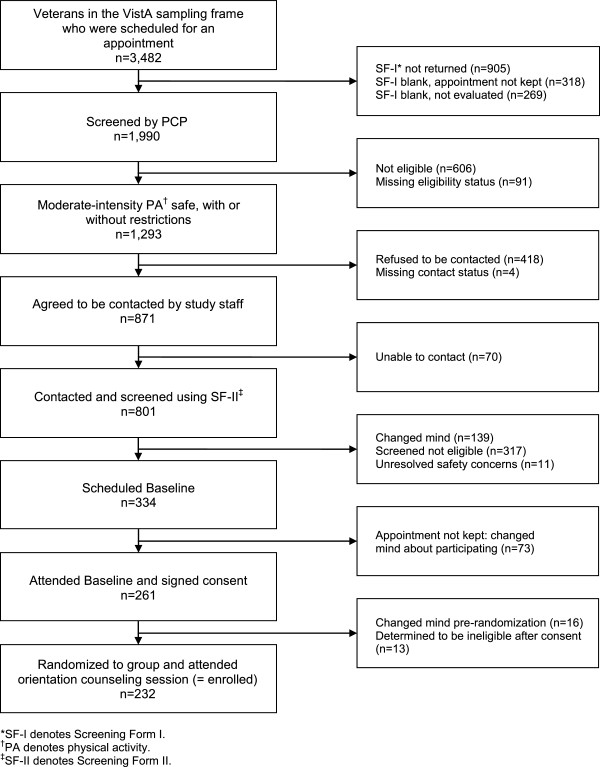
Results of VA-STRIDE screening and enrollment activities between 1 June 2010 and 31 April 2012.

The recruitment process from VistA prescreening through randomization is shown in Figure 1. We targeted those PCPs having the largest patient caseloads; 91 (36.1%) of the 252 PCPs providing care to veterans at VAPHS UD were approached and agreed to assist with recruitment. The total number of veterans receiving primary care at VAPHS UD during the recruitment period was 9,529. Of these, 6,329 (66.4%) received care from a participating provider. Applying the initial prescreening criteria for BMI, absence of exclusionary ICD-9 codes, and having at least 1 VAPHS UD visit in the prior 12 months, 3,482 (36.5%) potentially-eligible veterans who had scheduled appointments during the recruitment period were identified through the electronic screening of the medical records.

Overall, the majority of the PCPs deemed the intervention safe for their patients to participate with or without some restrictions (Table 1). Veterans were excluded if their PCPs did not specify whether or not they could participate safely (n = 91, 4.6%). No further screening occurred for the four cases (0.2%) for which the PCP did not indicate the veteran’s willingness to be contacted.

**Table 1:** Restrictions identified by primary care providers (PCPs) for veterans permitted to participate in moderate-intensity physical activity

**PCP-identified restrictions**	**No. (n = 397)**	**%**
Avoid vigorous activity	217	54.7
Avoid heat	78	19.6
Avoid cold temperatures	77	19.4
Non-weight bearing exercises, only	48	12.1
Avoid exercising alone	27	6.8
Prolong cooldown	25	6.3
Total	472^a^	

^a^Two or more restrictions were specified for some veterans.

As shown in Figure 1, research staff were able to contact 801 (92.0%) by telephone for completion of the SF-II. Safety concerns were identified in 54 (6.7%) and additional PCP clearance was sought and obtained for 43 (79.6%; see Table 2). As shown in Figure 1, 11 of these veterans (20.4%) were excluded from the study on the basis of safety, including 10 veterans for whom the PCP would require additional PA safety testing and 1 veteran for whom no additional safety information could be obtained from the PCP (Figure 1). Using as the denominator the 3,482 potentially eligible veterans who had scheduled appointments during the recruitment period, this recruitment approach resulted in a recruitment yield of 1 in 15.

**Table 2:** Safety exclusions requiring additional primary care provider (PCP) clearance prior to enrollment

**Patient-reported safety exclusions**	**No. (n = 54)**	**%**
Shortness of breath on mild exertion	22	40.7
Fast, irregular or extra heart beats	20	37.0
Balance problems due to dizziness	15	27.8
Chest pain or pressure with physical activity	14	25.9
Shortness of breath at rest	12	22.2
Chest pain or pressure at rest	8	14.8
Other medical problem that the veteran thought should preclude physical activity		
	5	9.3
History of fainting or passing out	2	3.7
Total	98^a^	

^a^Two or more safety exclusions were specified for some veterans.

The number of enrolled patients per month ranged from 1 to 27 (mean: 11). Initial recruitment was limited to a relatively small number of PCPs to ensure that the process was working well. Recruitment rates slowed substantially during the final 3 months of the enrollment period as eligible participants had been screened previously and there were insufficient numbers of new patients meeting study criteria. The study statisticians reviewed the implications of a sample size of 232 instead of the initially planned 300 and determined that continued enrollment would have minimal impact on study power. Consequently, recruitment was discontinued to preserve resources and ensure timely completion of the study.

## Discussion

Screening of patients by PCPs for clinical interventions is often time consuming and inefficient. The VistA prescreen in the VA-STRIDE study eliminated from consideration those veterans who were clearly ineligible or unlikely to benefit from the intervention, thereby minimizing the PCP screening burden. The recruitment approach also acknowledged time constraints of the PCP by giving them the option to 'override’ the SF-I (that is, leave the form blank) if they were too busy. The decision to have the patient, rather than the PCP, return the SF-I was based upon advice received from the medical director and staff of the primary care clinic. Because all veterans are required to return paperwork at checkout from the clinic, the investigators were advised to modify an existing procedure, rather than introduce another new procedure for busy PCPs. The only clinical judgments required of the PCPs were, first, whether or not unsupervised moderate intensity PA was safe for their patient and, if so, whether any exercise precautions were appropriate. The recruitment protocol did not rely on PCPs remembering to screen for the study but, rather, screening was triggered by the presence of a brightly-colored SF-I included with the veteran’s paperwork reviewed at the start of the clinic visit. To avoid overwhelming PCPs, in the early days of the study we initially capped the number of SF-Is they were asked to complete. VA-STRIDE had an office in VAPHS and our presence in the primary care clinic served as a reminder to PCPs to complete and return SF-Is.

The reasons for the patient’s return of a blank SF-I form, or the patient not returning the form at all, are unknown. We can speculate that a blank form indicated that the PCP was not able to complete the screening during the visit. Not returning the form, whether completed or not, could indicate that veterans chose to remove themselves from consideration for the study by choosing not to return their forms.

In a study by Embi *et al.*, which examined the use of electronic medical record auto alerts to recruit participants into a type 2 diabetes clinical trial, 90% of the auto alerts were dismissed [4]. A follow-up survey to providers in the Embi *et al*. study indicated that lack of time was the primary reason for dismissing auto alerts [14]. Additionally, lack of information on the clinical trial was a major reason for dismissing auto alerts in that study. Heinemann *et al*. also used electronic alerts to notify providers that their patients might be eligible for their study, and these were dismissed 68% of the time [11]. The 37% dismissal rate we observed in our study may have been aided by several factors: (1) VistA prescreening reduced burden (that is, Embi and Heinemann alerts were dismissed more often because providers were asked to evaluate more ineligible patients), (2) meetings of study PI and research staff with the PCPs to establish the relevance of PA research to the health of their patients, and (3) having the medical director of the clinic serve as the leading advocate for the study. This likely reduced the number of PCPs who were unaware of, or indifferent to, the study’s relevance to their patients.

## Conclusions

Using a semiautomated screening approach that combined an electronically-derived sampling frame with paper and pencil prescreening by PCPs and research staff, a high proportion of veterans potentially eligible for the VA-STRIDE study were screened by their PCPs. Methods to limit PCP burden, tailoring recruitment procedures to the operation of the clinic, and involvement of a clinical leader are key to the success of recruiting from a clinical practice setting.

## Abbreviations

BMI: Body mass index; IRB: Institutional review board; PA: Physical activity; PCPs: Primary care providers; PI: Principal investigator; SF-I: Screening form I; SF-2: Screening form II; VAPHS: VA Pittsburgh Healthcare System; UD: University drive division primary care clinic; VistA: Veterans health information systems and technology architecture.

## Competing interests

The authors declare that they have no competing interests.

## Authors’ contributions

MSH drafted the manuscript and oversaw its development. LJH contributed to designing the recruitment strategy, developing the data tracking system, and editing the manuscript. MAB contributed to designing the recruitment strategy, developing the data tracking system, recruiting participants, and editing the manuscript. MKM contributed to developing the data tracking system and editing the manuscript. ARS contributed to developing the data tracking system and editing the manuscript. SG contributed to developing the data tracking system and reviewing the manuscript. RAS contributed to designing the study and editing the manuscript. KHB contributed to designing the study and editing the manuscript. BHM contributed to designing the study and editing the manuscript. JTC contributed to designing the study and editing the manuscript. AMK contributed to designing the study and editing the manuscript. DTK contributed to designing the recruitment strategy, recruiting participants, and reviewing the manuscript. MAS designed the study, obtained funding, served as principal investigator, and edited the manuscript. All authors read and approved the final manuscript.

## Supplementary Material

Additional file 1 Screening form I (SF-I). Click here for file

Additional file 2 Screening form II (SF-II). Click here for file
