# Supplementary material for: Recruitment of veterans from primary care into a physical activity randomized controlled trial: the experience of the VA-STRIDE study
Source: Trials. 2014 Jan 7;15:11. doi: 10.1186/1745-6215-15-11 (PMC3923270; doi:10.1186/1745-6215-15-11)
Supplement: Additional file 1 — Screening form I (SF-I). [file 1745-6215-15-11-S1.docx]

**Appendix A. Screening Form I (SF-I)**

**VA-STRIDE Study Screening Form I**

**THREE-STEP ELIGIBILITY SCREENING PROCESS**

| **STEP 1. PCP Assessment:** **Given your knowledge of this patient, can this Veteran SAFELY do progressive, unsupervised physical activity at home** (regardless of his/her interest in doing so)**?** Participants may be asked to work up gradually to 30 minutes of moderate physical activity (e.g., brisk walking, swimming) on most days of the week. **Please check box 0, 1, or 2, for safety, below.** | | | | |
| --- | --- | --- | --- | --- |
| **NO / NOT SURE** 🡪 **0** □ **END of screening:** **SKIP to STEP 3.**  **YES** 🡪 **1** □ Permitted with no restrictions.  🡪 **2** □ Permitted with restrictions (check all that apply):  Non-weight bearing exercises □ Avoid vigorous activity □  Prolong cool down □ Avoid cold temperatures □  Avoid exercising alone □ Avoid heat □  Other (Specify): | | | | |
| **STEP 2. Is the Veteran willing to talk to a VA-STRIDE staff person about the study?**  **Yes □ _1_ No □ _0_**  *(Give the attached yellow handout to the patient.)* | | | | |
| **STEP 3. Please sign this form and give to patient along with the encounter form to return at check-out desk.**  **ALL screening forms, whether completed or not, must be collected by the research staff at the end of each day.**  **Thank you.** | | | | |
| PCP Signature: | Date: | | | |
| PCP Print Name: | |  |  |  |
