# Supplementary material for: Recruitment of veterans from primary care into a physical activity randomized controlled trial: the experience of the VA-STRIDE study
Source: Trials. 2014 Jan 7;15:11. doi: 10.1186/1745-6215-15-11 (PMC3923270; doi:10.1186/1745-6215-15-11)
Supplement: Additional file 2 — Screening form II (SF-II). [file 1745-6215-15-11-S2.docx]

**Appendix B. Screening Form II (SF-II)**

**VA-STRIDE Study Screening Form II**

To be completed via interview by VA-STRIDE Study Staff: In person □ Telephone □

Unable to contact for screening □ Patient changed mind about wanting to participate □

| **A. Exclusion Criteria:** | YES | NO |
| --- | --- | --- |
| 1. Are you able to walk the length of a football field (360 feet or 120 yards)? | □ _1_ | □ _0_ |
| 2. Do you require a cane, walker, or other device to help you walk (i.e., can’t walk without)? | □ _1_ | □ _0_ |
| 3a. Do you usually participate in regular physical activity similar to a brisk walk?  *By “brisk” we mean activities that require you to breathe a bit harder than usual and could include weight lifting, brisk walking, running, aerobic dance, swimming, playing sports, or physical labor associated with a job. For the activity to be counted, it needs to be for at least 10 continuous minutes without stopping.* | □ _1_  Ask Q3b. | □ _0_  Skip to Q4. |
| 3b. Do you participate in this sort of brisk physical activity for at least 60 minutes each week? | □ _1_ | □ _0_ |
| 4. Do you currently reside in a nursing home, personal care home, or other institution (e.g., prison)? | □ _1_ | □ _0_ |
| 5. Do you have difficulty reading printed material in English (e.g., a newspaper)? | □ _1_ | □ _0_ |
| 6. Do you plan to relocate out of the VA Pittsburgh Healthcare System service area within the next 12 months? | □ _1_ | □ _0_ |
| 7. Are you able and willing to travel to the University Drive VA or Highland Drive VA on 4 occasions over the next 12 months for study visits? | □ _1_ | □ _0_ |
| 8. Are you a VA Pittsburgh Healthcare System employee (include WOC employees)? | □ _1_ | □ _0_ |
| 9. Are you currently a participant in an intervention research study? | □ _1_ | □ _0_ |
| *Veterans with responses in any of the shaded boxes are to be thanked for their time and informed they are not eligible for the study. Continue with Safety Questions only for those Veterans who are eligible.* | | |
| **B. Safety Questions:** | YES | NO |
| 1. Do you ever experience chest pain or pressure at rest? | □ _1_ | □ _0_ |
| 2. Do you ever experience chest pain or pressure with physical activity? | □ _1_ | □ _0_ |
| 3. Do you ever experience shortness of breath at rest? | □ _1_ | □ _0_ |
| 4. Do you ever experience shortness of breath brought on by mild exertion? | □ _1_ | □ _0_ |
| 5. Do you ever faint or pass-out? | □ _1_ | □ _0_ |
| 6. Do you have fast, irregular or extra heart beats? | □ _1_ | □ _0_ |
| 7. Do you have trouble with your balance because of dizziness? | □ _1_ | □ _0_ |
| 8. Do you know of any other medical reason why you should not do physical activity?  If yes, please explain: | □ _1_ | □ _0_ |
| *Veterans who respond “yes” to any of the safety questions are to be informed that their PCP will be consulted before enrollment is pursued.* | | |
